# Efficacy and safety of apatinib in patients with recurrent or metastatic head and neck squamous cell carcinoma: a retrospective multi-center study

**DOI:** 10.1038/s41598-022-20272-x

**Published:** 2022-10-31

**Authors:** Zijing Liu, Zhuangzhuang Zheng, Lihua Dong, Xiao Guo, Xiaojing Jia, Jianfeng Wang, Lingbin Meng, Xiangyan Cui, Xin Jiang

**Affiliations:** 1 Jilin Provincial Key Laboratory of Radiation Oncology and Therapy, The First Hospital of Jilin University, Changchun, 130021 China; 2 Department of Radiation Oncology, The First Hospital of Jilin University, Changchun, 130021 China; 3 NHC Key Laboratory of Radiobiology, School of Public Health of Jilin University, Changchun, 130021 China; 4 Department of Radiation Oncology, Jilin Cancer Hospital, Changchun, 130000 China; 5 Department of Radiation Oncology, The Second Hospital of Jilin University, Changchun, 130021 China; 6 Department of Radiation Oncology, China-Japan Union Hospital of Jilin University, Changchun, 130031 China; 7 Department of Hematology and Medical Oncology, Moffitt Cancer Center, Tampa, FL 33612 USA; 8 Department of Otolaryngology-Head and Neck Surgery, The First Hospital of Jilin University, Changchun, 130021 China

**Keywords:** Cancer, Molecular medicine, Oncology

## Abstract

Apatinib is a novel antiangiogenic agent that targets vascular endothelial growth factor 2. The aim of our study was to explore the efficacy and safety of apatinib in the treatment of patients with recurrence or metastasis (R/M) inoperable head and neck squamous cell carcinoma (HNSCC). This multi-center retrospective study analyzed 53 cases of recurrent or metastatic inoperable HNSCC who had progressed or recurred after undergoing standard radiotherapy, chemotherapy, and immunotherapy treated with apatinib from March 2017 to August 2021. Patients continued apatinib until the time of disease progression or onset of intolerable adverse events. The primary endpoint was progression-free survival (PFS), and the secondary endpoints were overall survival (OS), objective response rate (ORR), and disease control rate (DCR) and incidence of adverse events. Univariable and multivariable analyses were performed to determine prognostic factors. The main adverse events were counted, and the severity of the adverse reactions was evaluated. Fifty-three patients with recurrent or metastatic inoperable R/M HNSCC who had progressed or recurred after standard radiotherapy, chemotherapy, and immunotherapy were included. The ORR was 15.1%, and the DCR was 86.8%. The median PFS was 4.4 months (95% confidence interval [CI] 3.7–5.0 months) and the median OS was 6.6 months (95% CI 5.3–7.9 months). The number of apatinib lines was an influencing factor for both PFS and OS, and the Eastern Cooperative Oncology Group (ECOG) score, tumor differentiation, and apatinib duration were only the influencing factors for OS. Of these, only the ECOG score was an independent predictor of OS. The most common adverse reactions were hypertension (39.6%), hand-foot syndrome (32.1%), fatigue (32.1%), oral ulcers (28.3%), and nausea and vomiting (20.8%). Most adverse reactions were grade 1 or 2. Apatinib mesylate has good efficacy for recurrent/metastatic inoperable HNSCC as second-line and above-line treatment. ECOG score was an independent prognostic factors of OS in patients who were treated with apatinib. In addition, the adverse effects of apatinib mesylate were relatively mild.

## Introduction

Head and neck tumors are the seventh most common malignant tumors worldwide, accounting for about 6% to 7% of systemic malignancies, and most are squamous cell carcinomas^1^. Local recurrence and distant metastasis occur in approximately 50% of patients with head and neck cancer^2^, and are the main reasons for treatment failure. Platinum combined with 5-fluorouracil (5-FU) was the standard treatment for patients with relapsed or metastatic inoperable head and neck squamous cell carcinoma (HNSCC); the introduction of cetuximab to this combination treatment was shown to significantly improve the median overall survival (OS) and median progression-free survival (PFS) in these patients^3^. However, the standard first-line treatment has changed over time, and immunotherapy is now a first-line choice^4^.

In recent years, the role of immunotherapy in patients with relapsed or metastatic inoperable HNSCC has become an important focus of clinical research. Pembrolizumab, a PD-1 inhibitor, has greatly improved the survival of patients with relapsed or metastatic inoperable HNSCC in the form of single agent or combined chemotherapy (KEYNOTE-048). Pembrolizumab is an effective first-line treatment for patients with high PD-L1 expression^5^. According to the latest National Comprehensive Cancer Network (NCCN) guidelines, single-agent pembrolizumab is the recommended first-line treatment for patients with PD-L1 expression and CPS ≥ 20, while pembrolizumab and platinum-based chemotherapy is recommended for patients with CPS ≥ 1. However, for recurrent or metastatic inoperable HNSCC with low CPS expression, current evidence suggests that pembrolizumab is not significantly more effective than standard cetuximab, platinum, and 5-FU combination therapy. Therefore, for the patients with low CPS expression, standard cetuximab, platinum and 5-FU combination therapy is still the first-line standard treatment^6,7^. In cases of relapse after platinum therapy, nivolumab or pembrolizumab are appropriate second-line treatments in patients who have not received immunotherapy.

Anti-angiogenesis therapy has become the standard treatment for a variety of malignant tumors, including colon adenocarcinoma, non-small cell lung cancer, renal cell carcinoma, cervical cancer, glioblastoma multiforme, ovarian cancer, and hepatocellular carcinoma, soft tissue sarcoma, and gastric cancer^8^. Bevacizumab was shown to have a significant inhibitory effect in HNSCC tumor xenografts in mice^9^. In humans, a phase II study of bevacizumab combined with pemetrexed showed a comparable treatment effect to the standard regimen of platinum, 5-FU, and cetuximab, and the adverse reactions were well tolerated^4,10^. In a phase III randomized trial of chemotherapy with or without bevacizumab for patients with recurrent or metastatic head and neck cancer, although the median OS difference between bevacizumab combined with chemotherapy and chemotherapy alone was not statistically significant, the median PFS increased from 4.3 months to 6.0 months (HR 0.71; p = 0.0012) and the overall response rate (ORR) increased from 24.5% to 35.5% (p = 0.013) after adding bevacizumab to chemotherapy^11^. However, patients in the bevacizumab group experienced more bleeding-related adverse reactions. Therefore, anti-angiogenic drugs are not currently approved for the treatment of HNSCC by the Food and Drug Administration. The application of anti-angiogenesis therapy in recurrent/metastatic HNSCC still needs further exploration.

Apatinib, a new type of small molecule inhibitor targeting VEGFR-2, can selectively target ATP binding sites in cells and has five times the binding capacity of sunitinib^12^. Apatinib can effectively inhibit the kinase activity of VEGFR-2, c-kit and c-src, and inhibit the phosphorylation of VEGFR-2, c-kit and PDGFRβ to inhibit tumor growth, reduce microvessel density and promote tumor cell apoptosis in mice. In human tumor xenograft models, the combined treatment of apatinib and chemotherapy has the lowest FDG uptake, and its inhibitory effect on tumor growth has been confirmed^12,13^. Apatinib combined with carrelizumab in the treatment of advanced osteosarcoma, triple-negative breast cancer, advanced cervical cancer, advanced esophageal squamous cell carcinoma, and extensive-stage small cell lung cancer have obtained very good curative effects with acceptable safety^14–18^. In a previously published clinical study on the efficacy and safety of single-agent apatinib for relapsed/metastatic nasopharyngeal carcinoma that had failed previous chemotherapy, the median OS and PFS were 16 (95% confidence interval [CI], 9.3–22.7) and 9 months (95% CI 5.2–12.8), respectively^19^. This is the valuable clinical data regarding apatinib in the treatment of recurrent/metastatic head and neck malignancies so far, but comprehensive clinical data are still lacking. To this end, the current study aimed to explore the efficacy and safety of apatinib in the treatment of recurrent or metastatic HNSCC.

## Materials and methods

### Patient eligibility

This retrospective study was approved by the Ethics Committee of the First Hospital of Jilin University.

Patients with recurrent or metastatic inoperable squamous cell carcinoma who progressed or recurred after undergoing standard radiotherapy, chemotherapy, and immunotherapy in accordance with the recommendations and guidelines of the NCCN were enrolled according to the following inclusion criteria: (1) age ≥ 18 years old, regardless of sex; (2) history of chemotherapy or molecular targeted therapy and evidence of R/M and according to the Response Evaluation Criteria in Solid Tumors (RECIST) 1.1 criteria; (3) Eastern Cooperative Oncology Group (ECOG) score 0–3; (4) acceptable baseline blood routine and biochemical results: hemoglobin ≥ 80 g/L, platelets ≥ 80 × 10^9^/L, alanine transaminase and aspartate aminotransferase ≤ 2.5 times the upper limit of normal (≤ 5 times the upper limit of normal for patients with liver metastases) serum total bilirubin ≤ 1.5 times the upper limit of normal, serum creatinine ≤ 1.5 times the upper limit of normal, and serum albumin ≥ 30 g/L; (5) at least one measurable lesion determined by the RECIST 1.1 criteria; (6) completed at least one cycle (4 weeks) of apatinib; and (7) surgery cannot be performed due to absolute or relative contraindications such as poor general status or older age. Exclusion criteria were: (1) abnormal coagulation function, with bleeding tendency; (2) pregnant or lactating women; (3) concomitant diseases that seriously endanger the safety of patients or affect the completion of the study.

We collected 57 recurrent/metastatic inoperable HNSCC patients from March 2017 to August 2021 including nasopharyngeal squamous cell carcinoma and non-nasopharyngeal squamous cell carcinoma. Four patients were excluded due to lack of follow-up data. Table 1 shows the clinicopathological characteristics of patients at the beginning of apatinib treatment. Except for one case of cervical lymph node metastatic squamous cell carcinoma with unknown primary focus, the primary tumor types included in the study were all squamous cell carcinoma. The primary tumor sites included the oral cavity (4 cases of mouth floor cancer, 1 case of oral cancer, 5 cases of tongue cancer, 2 cases of gum cancer, 2 cases of tonsil cancer) (n = 14, 26.4%), pharynx (12 cases of nasopharyngeal cancer, 1 case of oropharyngeal cancer, 11 cases of hypopharyngeal cancer) (n = 24, 45.3%), larynx (n = 10, 18.9%), parotid gland (n = 1, 1.9%), maxillary sinus (n = 2, 3.8%), mandible (n = 1, 1.9%), and unidentified primary lesion (n = 1, 1.9%). Of the total study population, 26 patients had primary tumor progression, 11 had primary tumor progression with distant organ metastasis, and 16 had only distant organ metastasis. Of the 26 patients with primary tumor progression, 21 patients whose primary lesions progressed had progressed again after receiving treatment for the purpose of radical cure. Thirty-two patients received palliative care due to late tumor staging, poor patient status, or older age. Thirty-seven patients received apatinib monotherapy due to intolerance or refusal of chemotherapy, of which six patients were combined with local radiotherapy. Sixteen patients received apatinib in combination with chemotherapy, two of whom received concurrent radiotherapy.

**Table 1:** Clinical and treatment characteristics of 54 recurrent metastatic head and neck squamous cell carcinom patients who received apatinib.

Characteristics	Value
**Age**	
Median	59 years
Range	28–85 years
**Sex**	
Male	42 (79.2%)
Female	11 (20.8%)
**ECOG performance status**	
0–1	30 (56.6%)
2–3	23 (43.4%)
**Tumor type, no (%)**	
Nasopharyngealsquamous carcinoma	12 (22.6%)
Hypopharyngeal squamous carcinoma	11 (20.8%)
Laryngeal squamous carcinoma	10 (18.9%)
Tongue squamous carcinoma	5 (9.4%)
Mouth floor squamous carcinoma	4 (7.5%)
Unknown primary squamous carcinoma	12 (22.6%)
**Metastasis sites of involvement**	
Lung	12 (22.6%)
Bone	4 (7.5%)
Liver	3 (5.7%)
Brain	2 (3.8%)
Non metastasis	26 (49.1%)
**Differentiation**	
Poorly differentiated and undifferentiated	19 (35.8%)
Moderately differentiated and well differentiated	27 (50.9%)
Unknown	7 (13.2%)
**Primary tumor progression and/or metastasis**	
Primary tumor progression	26 (49.1%)
Primary tumor progression and metastasis	11 (20.8%)
Metastasis	16 (30.2%)
**Intent of treatment at diagnosis**	
Curative	21 (39.6%)
Palliative	32 (60.3%)
**Radiotherapy of the primary tumor**	
Yes	47 (88.7%)
No	6 (11.3%)
**Line of apatinib**	
2 line	27 (50.9%)
3 line and further line	26 (49.1%)
**Combination therapy**	
Apatinib monotherapy	31 (58.5%)
Apatinib combined with radiotherapy	6 (11.3%)
Apatinib combined with chemotherapy	14 (26.4%)
Apatinib combined with radiotherapy and chemotherapy	2 (3.8%)

*ECOG* Eastern Cooperative Oncology Group.

### Treatment and dose adjustment

All patients were treated with apatinib. In principle, patients with recurrent or metastatic inoperable head and neck squamous cell carcinoma who are resistant to first-line or further systemic therapy should receive apatinib combination therapy. Patients treated with single-agent apatinib were those who could not tolerate chemotherapy or who refused chemotherapy. For patients with indications for radiotherapy, we administered palliative radiotherapy to recurrent or metastatic disease in addition to systemic therapy. Apatinib was initially administered at a dose of 250 mg per day for four weeks and adjusted according to clinical need. In some cases, the dose was increased to 500 mg based on patient tolerance and requirements. In cases of serious adverse events, treatment was either interrupted, reduced to 125 mg per day, or permanently stopped. Combination treatment with apatinib and other medication was at the discretion of the treating physician and based upon the patient's general physical condition. Patients were followed up until disease progression, death, discontinuation of treatment due to intolerable toxicity, or until August 22, 2021, whichever occurred first.

The doses of apatinib were 250 mg in 47 patients, 125 mg in 1 patient, and 500 mg in 5 patients.

### Efficacy and safety assessment

The primary analytical endpoint was PFS, and the secondary analytical endpoints were OS, disease control rate (DCR), objective response rate (ORR) and incidence of adverse events. PFS was defined as the time from the start of apatinib treatment to disease progression or death, whichever occurred first. OS was defined as the time from the initiation of apatinib treatment to death from any cause.

Tumor response was evaluated by radiologists and oncologists according to the RECIST 1.1 criteria and based on imaging data or clinical manifestations. Tumor response includes complete response (CR), partial response (PR), stable disease (SD), and progressive disease (PD). ORR was defined as the proportion of patients with CR or PR. DCR was defined as the sum of the ORR and SD. Toxicity was evaluated according to the National Cancer Institute Common Toxicity Standards for Adverse Events Version (CTCAE) 4.0, and determined based on the patient's medical history and laboratory test results or telephone follow-up.

According to the criteria above, the median PFS and median OS of nasopharyngeal squamous cell carcinoma and non-nasopharyngeal squamous cell carcinoma were calculated respectively.

### Statistical analysis

Quantitative data are expressed as median (range) or number of patients (percentage). Survival analysis was performed using the Kaplan–Meier method. The log-rank test and Cox Regression was used for comparison. Exploratory univariable analysis was performed using the log-rank test and Cox regression. Included the following variables: age, sex, ECOG score, primary tumor site, primary tumor stage, metastatic lymph node stage, metastasis, tumor differentiation, line of apatinib treatment, duration of apatinib treatment, and the combined or non-combined administration of apatinib. The variables with p < 0.05 from univariable analysis were included in Cox model for multivariable analysis. We use frequency counts and percentages to summarize adverse events (AE). SPSS version 26.0 (SPSS Inc., Chicago, IL, USA) and GraphPad Prism 8.0.1 (GraphPad Software, San Diego, California, USA) was used for statistical analysis. *P* < 0.05 was considered statistically significant.

### Statement

This study was conducted in accordance with the Declaration of Helsinki. All authors confirm that all methods were carried out in accordance with relevant guidelines and regulations. Written informed consent was obtained from each patient and/or their legal guardians.

### Institutional Review Board Statement

 The study was approved by the Ethics Committee of the First Hospital of Jilin University (protocol code: 2016-459, date of approval: 10 December 2016).

## Results

### Efficacy of apatinib in the treatment of R/M HNSCC

Reasons for stopping apatinib included PD (n = 29, 54.7%), death (n = 13, 24.5%), and adverse reactions (n = 2, 3.8%). In 3 patients (5.7%), the reason for stopping apatinib was unknown. Of the 53 patients with evaluable treatment response, 8 patients (15.1%) had PR, 38 patients (71.7%) had SD, 7 patients (13.2%) had PD, and no patient achieved CR. The DCR was 86.8% and the ORR was 15.1% (Table 2).

**Table 2:** Objective response rate (ORR) and disease control rate (DCR) of apatinib in recurrent/metastatic inoperable head and neck squamous cell carcinoma patients.

Tumor response	N (%)
CR	0
PR	8 (15.1%)
SD	38 (71.7%)
PD	7 (13.2%)
ORR(CR + PR)	15.1%
DCR(CR + PR + SD)	86.8%

Responses were graded using the RECIST criteria v1.1.

*RECIST* Response Evaluation Criteria in Solid Tumors, *CR* complete response, *PR* partial response, *SD* stable disease, *PD* progressive disease.

**Figure 1:**
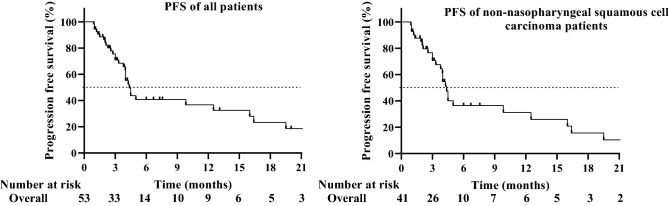
Kaplan–Meier graph for progression-free survival (PFS) of all patients (n = 53) and non-nasopharyngeal squamous cell carcinoma patients (n = 41): median PFS of all people was 4.4 months (95% CI 3.71–5.03 months). Median PFS of non-nasopharyngeal squamous cell carcinoma patients was 4.4 months (95% CI 3.93–4.81).

At the end of the follow-up, the median PFS was 4.4 months (95% CI 3.71–5.30), and the 1-year PFS was 17.0%. The six patients currently taking apatinib showed no evidence of disease progression at the last follow-up. Among them, for patients with nasopharyngeal carcinoma, no median PFS data were available. For patients with non-nasopharyngeal carcinoma, the median PFS was 4.4 months (95% CI 3.93–4.81), which was the same as the median PFS of all patients. In R/M HNSCC patients, a stratified analysis was performed using different concurrent treatment modalities. The mPFS was 4.4 months with apatinib monotherapy, 3.3 months with apatinib plus radiotherapy, and 4.0 months with apatinib plus chemotherapy. The mPFS of apatinib combined with radiotherapy was not significantly prolonged compared with that of monotherapy (P = 0.69). The mPFS of apatinib combined with radiotherapy was not significantly prolonged compared with that of monotherapy (P = 0.75). The Kaplan–Meier analysis of PFS is shown in Fig. 1.

**Figure 2:**
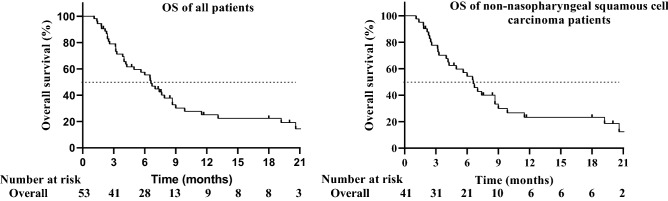
Kaplan–Meier graph for progression-free survival (OS) of all patients (n = 53) and non-nasopharyngeal squamous cell carcinoma patients (n = 41): Median OS of all people was 6.6 months (95% CI 5.30–7.90 months). Median OS of non-nasopharyngeal squamous cell carcinoma patients was 6.6 months (95% CI 5.06–8.14).

At the end of the follow-up, the median OS was 6.6 months (95% CI 5.30–7.90 months), and the 1-year OS was 17.0%. Among them, for patients with nasopharyngeal carcinoma, the median OS was 6.5 months (95% CI 0.90–12.10). For patients with non-nasopharyngeal carcinoma, the median OS was 6.6 months (95% CI 5.06–8.14), which was the same as the median OS of all patients. In R/MHNSCC patients, a stratified analysis was performed using different concurrent treatment modalities. The mOS for patients receiving apatinib plus chemotherapy and monotherapy was 6.5 months and 5.6 months, respectively. However, the mOS of apatinib plus chemotherapy was not significantly prolonged compared with monotherapy (P = 0.73). The mOS in patients who received apatinib plus radiotherapy was not available. The Kaplan–Meier analysis of OS is shown in Fig. 2.

### Prognostic factors affecting OS and PFS

**Figure 3:**
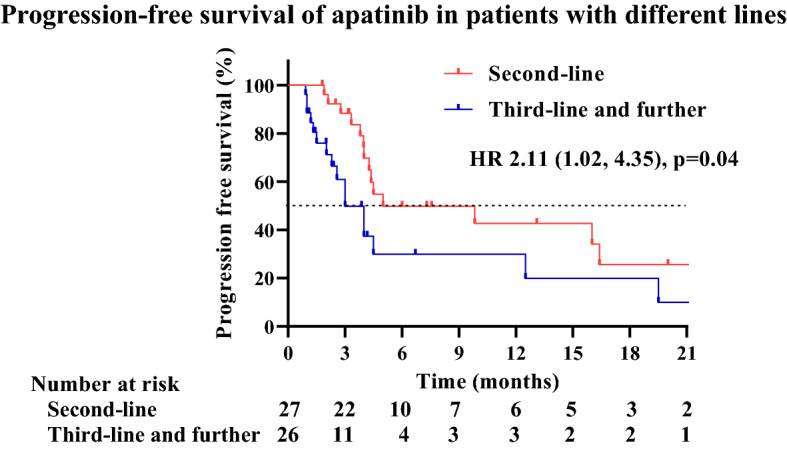
In univariable analysis, comparison of PFS between patients treated with apatinib as the second-line medication with the third-line and further line medication. The median PFS was 5.0 months (95% CI 0.00–11.82) for treatment with apatinib as the second-line medication versus 3.0 months (95% CI 1.68–4.32) for treatment with apatinib as the third-line and further line medication (p = 0.04).

We compared the survival outcomes of the different prognostic factors using univariable and multivariable analyses. Univariable analysis of PFS showed that only the number of lines of apatinib may impact the PFS of patients. The mPFS for patients taking apatinib as second- and third-line was 5.0 months and 3.0 months (HR = 2.11; 95% CI 1.02–4.35; P = 0.04) (Fig. 3). The mPFS of patients of different genders (male and female) treated with apatinib was 4.5 months and 4.4 months, respectively (HR = 0.92; 95% CI 0.37–2.26; P = 0.85). The mPFS of patients of different ages (< 65 years vs ≥ 65 years) treated with apatinib was 4.5 months and 4.3 months, respectively (HR = 0.84; 95% CI 0.37–1.89; P = 0.67). Patients with ECOG scores of 0–1 and 2–3 had mPFS of 4.5 and 4.0 months with apatinib, respectively (HR = 1.30; 95% CI 0.61–2.76; P = 0.50). The mPFS of patients taking apatinib ≤ 60 days and > 60 days was 4.4 months and 4.5 months, respectively (HR = 1.11; 95% CI 0.54–2.29; P = 0.789) (Table 3).

**Table 3:** Univariable progression free survival results for patient characteristics.

	No. of events/patients	Univariable	
		PFS (median, 95% CI)	*p* value
**Gender**			
Male	24/42	1	0.85
Female	6/11	0.92 (0.37, 2.26)	
**Age**			
< 65	22/35	1	0.67
≥ 65	8/18	0.84 (0.37, 1.89)	
**ECOG**			
0–1	19/30	1	0.50
2–3	11/23	1.30 (0.61, 2.76)	
**Primary tumor site**			
Nasopharyngeal carcinoma	4/12	1	0.16
Non-nasopharyngeal carcinoma	26/41	2.14 (0.74, 6.14)	
**T-stage**			
T0–T2	13/21	1	0.60
T3–T4	11/25	0.80 (0.36, 1.81)	
Unknown	6/7	–	
**N-stage**			
N0–N1	7/14	1	0.83
N2–N3	17/32	0.91 (0.37, 2.19)	
Unknown	6/7	–	
**Metastatic tumor**			
Yes	14/27	0.57 (0.27, 1.19)	0.13
No	15/24	1	
Unknown	1/2	–	
**Differentiation**			
Poorly differentiated and undifferentiated	6/19	0.68 (0.27, 1.72)	0.41
Moderately differentiated and well differentiated	18/27	1	
Unknown	6/7	–	
**Duration of medication**			
≤ 60 days	13/26	1.11 (0.54, 2.29)	0.79
> 60 days	17/27	1	
**Line of apatinib**			
2 line	14/27	1	0.04
3 line and further line	16/26	2.11 (1.02, 4.35)	
**Combination therapy**			
Apatinib monotherapy	17/31	1	
Apatinib combined with radiotherapy	3/6	0.82 (0.24, 2.80)	0.75
Apatinib combined with chemotherapy	8/14	1.25 (0.54, 2.90)	0.61
Apatinib combined with radiotherapy and chemotherapy	2/2	2.43 (0.55, 10.71)	0.24

**Figure 4:**
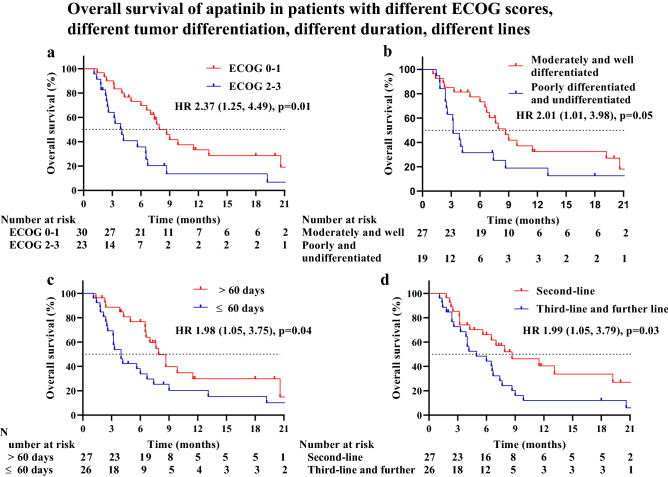
In univariable analysis, (**a**) comparison of OS between patients with an initial ECOG score of 0–1 and an ECOG score of 2–3. The median OS was 8.7 months (95% CI 6.85–10.49) for patients with an initial ECOG score of 0–1 versus 4.0 months (95% CI 2.73–5.27) for patients with an initial ECOG score of 2–3 (p = 0.01). (**b**) Comparison of OS between patients with moderately differentiated and well-differentiated tumors and poorly differentiated and undifferentiated tumors. The median OS was 8.7 months (95% CI 6.68–10.67) for patients with moderately differentiated and well-differentiated tumors versus 3.2 months (95% CI 2.21–4.20) for patients with poorly differentiated and undifferentiated tumors (p = 0.05). (**c**) Comparison of OS between patients treated with apatinib for more than 60 days and within 60 days. The median OS was 7.9 months (95% CI 6.18–9.63) for patients treated with apatinib for more than 60 days versus 4.0 months (95% CI 2.79–5.21) for patients treated with apatinib for within 60 days (p = 0.04). (**d**) Comparison of OS between patients treated with apatinib as the second-line medication with the third-line and further line medication. The median OS was 8.7 months (95% CI 3.08–14.26) for treatment with apatinib as the second-line medication versus 5.0 months (95% CI 1.73–8.21) for treatment with apatinib as the third-line and further line medication (p = 0.03).

Univariable analysis of OS showed that ECOG score, tumor differentiation, duration of apatinib, and number of lines of apatinib had impacts on it. Patients with an ECOG score of 0–1 (8.7 months) treated with apatinib had significantly longer mOS compared with patients with an ECOG score of 2–3 (4.0 months) (HR = 2.37; 95% CI 1.25–4.49; P = 0.01) (Fig. 4a). Patients with moderately or well-differentiated tumors (8.7 months) who received apatinib had significantly longer mOS compared with patients with poorly differentiated or undifferentiated tumors (3.2 months) (HR = 2.01; 95% CI 1.01–3.98; P = 0.05) (Fig. 4b). Compared with patients who received apatinib for ≤ 60 days (4.0 months), patients who received apatinib for > 60 days (7.9 months) had significantly longer mOS (HR = 1.98; 95% CI 1.05–3.75; P = 0.04) (Fig. 4c). Patients taking apatinib as second-line (8.7 months) had significantly longer mOS than third-line and above (5.0 months) (HR = 1.99; 95% CI 1.05–3.79; P = 0.03) (Fig. 4d). In addition, the mOS of male and female patients treated with apatinib was 6.5 months and 7.6 months, respectively (HR = 0.72; 95% CI 0.31–1.64; P = 0.43). The mOS for patients < 65 years and ≥ 65 years who received apatinib were 7.0 months and 4.2 months, respectively (HR = 1.44; 95% CI 0.74–2.79; P = 0.28) (Table 4).

**Table 4:** Univariable and multivariable overall survival results for patient characteristics.

	No. of events/patients	Univariable		Multivariable	
		OS (median, 95% CI)	*p* value	Adjusted hazard ratio (95% CI)	*p* value
**Gender**					
Male	32/42	1	0.43		
Female	7/11	0.72 (0.31, 1.64)			
**Age**					
< 65	25/35	1	0.28		
≥ 65	14/18	1.44 (0.74, 2.79)			
**ECOG**					
0–1	20/30	1	0.01	1	0.01
2–3	19/23	2.37 (1.25, 4.49)		2.62(1.32,5.21)	
**Primary tumor site**					
Nasopharyngeal carcinoma	9/12	1	0.86		
Non-nasopharyngeal carcinoma	30/41	1.07 (0.51, 2.26)			
**T-stage**					
T0–T2	15/21	1	0.50		
T3–T4	19/25	1.27 (0.64, 2.50)			
Unknown	6/7	–			
**N-stage**					
N0–N1	10/14	1	0.72		
N2–N3	24/32	0.87 (0.42, 1.83)			
Unknown	6/7	–			
**Metastatic tumor**					
Yes	21/27	0.96 (0.51, 1.83)	0.91		
No	17/24	1			
Unknown	1/2	–			
**Differentiation**					
Poorly differentiated and undifferentiated	16/19	2.01 (1.01, 3.98)	0.05	1.21 (0.55, 2.62)	0.64
Moderately differentiated and well differentiated	18/27	1		1	
Unknown	5/7	–		–	
**Duration of medication**					
≤ 60 days	22/26	1.98 (1.05, 3.75)	0.04	1.72 (0.82, 3.61)	0.15
> 60 days	17/27	1		1	
**Line of apatinib**					
2 line	16/27	1	0.03	1	0.19
3 line and further line	23/26	1.99 (1.05, 3.79)		1.60 (0.79, 3.27)	
**Combination therapy**					
Apatinib monotherapy	22/31	1			
Apatinib combined with radiotherapy	3/6	0.64 (1.19, 2.15)	0.47		
Apatinib combined with chemotherapy	12/14	1.37 (0.68, 2.78)	0.38		
Apatinib combined with radiotherapy and chemotherapy	2/2	0.88 (0.21, 3.80)	0.87		

Multivariable analysis showed that ECOG score (HR = 2.62; 95% CI 1.32–5.21; P = 0.01) was an independent predictor of OS, and patients' status before apatinib administration had a direct impact on survival. However, tumor differentiation (HR = 1.21; 95% CI 0.55–2.62; P = 0.64), number of lines of apatinib treatment (HR = 1.60; 95% CI 0.79–3.27; P = 0.19), and duration of apatinib treatment (HR = 1.72; 95% CI 0.82–3.61; P = 0.15) were not independent predictors of OS (Table 4).

### Safety outcomes

All patients were included in the safety analysis set. Hypertension (39.6%), hand-foot syndrome (32.1%), fatigue (32.1%), oral ulcers (28.3%) and nausea and vomiting (20.8%) were the most common adverse events in this study (Table 5). The adverse events in most patients were all grade 1 or 2 according to the CTCAE 4.0. In most patients, hypertension was mild and effectively controlled with oral antihypertensive drugs. Hand-foot syndrome and fatigue did not significantly affect patients’ quality of life. Oral ulcers did not affect patients’ oral intake. Only 2 patients had grade 3/4 adverse reactions. One patient discontinued treatment due to severe oral ulcers, and one patient was admitted to hospital due to thrombocytopenia caused by severe bone marrow suppression.

**Table 5:** Major treatment-related adverse events, n (%).

Adverse events	Grade 1, n (%)	Grade 2, n (%)	Grade 3–4, n (%)	All, n (%)
**Non-haematological**				
Hypertension	7 (13.2%)	14 (26.4%)	0	21 (39.6%)
Fatigue	11 (20.8%)	6 (11.3%)	0	17 (32.1%)
Hand-foot syndrome	11 (20.8%)	6 (11.3%)	0	17 (32.1%)
Oral ulcer	7 (13.2%)	7 (13.2%)	1 (1.9%)	15 (28.3%)
Proteinuria	2 (3.8%)	1 (1.9%)	0	3 (5.7%)
Hypothyroidism	0	1 (1.9%)	0	1 (1.9%)
Hemorrhage	2 (3.8%)	1 (1.9%)	0	3 (5.7%)
Nausea and vomiting	9 (17.0%)	2 (3.8%)	0	11 (20.8%)
Elevated aminotransferase	1 (1.9%)	1 (1.9%)	0	2 (3.8%)
Hoarseness	1 (1.9%)	1 (1.9%)	0	2 (3.8%)
Constipate	4 (7.5%)	0	0	4 (7.5%)
Toothache	2 (3.8%)	1 (1.9%)	0	3 (5.7%)
Elevated creatinine	1 (1.9%)	0	0	1 (1.9%)
Gastrointestinal discomfort	1 (1.9%)	2 (3.8%)	0	3 (5.7%)
**Haematological**				
Anemia	0	3 (5.7%)	0	3 (5.7%)
Leukopenia	0	1 (1.9%)	0	1 (1.9%)
Thrombocytopenia	1 (1.9%)	2 (3.8%)	1 (1.9%)	4 (7.5%)

## Discussion

HNSCC is the sixth most common tumor and one of the most common malignant tumors^20^. About 50% of patients with recurrent/metastatic head and neck squamous cell carcinoma will relapse after first-line therapy, and these patients have a particularly poor prognosis^21^. The immune checkpoint inhibitors nivolumab and pembrolizumab are currently approved in some countries. However, some studies suggest that immunotherapy may be more suitable for first-line rather than second-line treatment^22^. The response rates of methotrexate, taxanes, afatinib, gefitinib and other drugs recommended in the current guidelines as second-line treatment are still very low, and the choice of second-line treatment drugs is still very limited. Apatinib is administered orally without the need for hospitalization or infusion pumps, which may improve patient compliance and economic benefits.

This study retrospectively analyzed the efficacy of apatinib, a small-molecule inhibitor targeting VEGFR-2, in the treatment of second-line or above inoperable R/M HNSCC. In non-nasopharyngeal R/MHNSCC patients, the mOS and mPFS of apatinib monotherapy were 5.6 months and 4.4 months. Compared with the second-line drugs recommended in the current NCCN guidelines, apatinib has insufficient survival benefits compared with nivolumab (mOS: 7.5 months) and pembrolizumab (mOS: 8.4 months), and is slightly lower than afatinib (mOS: 6.9 months), methotrexate (mOS: 6.4 months)^7,23,24^. However, single-agent apatinib was comparable to gefitinib monotherapy (mOS: 5.6 months)^25^. In addition, apatinib monotherapy had significantly longer mPFS compared with nivolumab and afatinib (mPFS were 2.0 months and 2.9 months, respectively)^23,24^. Comparing our results with recently published high-quality RCTs of second-line therapy, apatinib monotherapy resulted in lower mOS but significantly longer mPFS^26^. Compared with other anti-angiogenic drugs, the mPFS and mOS of apatinib monotherapy were significantly higher than those of sorafenib (mPFS: 1.8 months; mOS: 4.2 months), sunitinib (mPFS: 2 months; mOS: 3.4 months)^27,28^. This may be due to the higher inhibition rate of VEGFR-2 by apatinib than the latter two^29–31^. However, the above studies of anti-angiogenic drugs are limited to phase II clinical studies, and further phase III clinical studies are still needed for further exploration. As second-line and above treatment, the mOS of single-agent apatinib in nasopharyngeal carcinoma was 6.5 months, and no mPFS was obtained. The mOS of our study was much lower than the other study^19^. We compared patient baselines and treatment modalities with this study. First, the main reason is the limited number of patients we included. Secondly, the doses of apatinib given in their studies were all 500 mg, but only 2 patients with nasopharyngeal carcinoma received a dose of 500 mg of apatinib in our study.

Our study showed that ORR was 15.1% and DCR was 86.8%, which was similar to axitinib (ORR: 6.7%; DCR: 76.7%)^32^. Vascular normalization can reduce the pressure of tissue fluid to facilitate the entry of oxygen and drugs^33^. This suggests that apatinib may be combined with radiotherapy and other treatments to improve efficacy. However, our study showed that radiotherapy plus apatinib or chemotherapy plus apatinib did not significantly increase mPFS and mOS compared with single-agent apatinib. We believe that this may be due to the fact that patients enrolled on single-agent apatinib tended to have more stable disease and slower progression than patients on combination therapy. Patients treated with apatinib monotherapy had higher baseline levels at the start of treatment.

In univariable analysis, there were no significant differences in mPFS and mOS by gender and age. Patients with moderately or well-differentiated tumors had significantly longer mOS compared with patients with poorly differentiated or undifferentiated tumors. Patients with ECOG scores 0–1 had significantly longer mOS than those with ECOG scores 2–3, but different ECOG scores had no significant effect on mPFS. This suggests that patients with good general status are more likely to benefit from apatinib. In all patients, mPFS and mOS were significantly longer in second-line than in third- and above-line. In addition, patients treated with apatinib for more than 60 days had significantly longer mOS than those within 60 days, suggesting that early treatment of R/M HNSCC with apatinib may improve efficacy. Unfortunately, although univariable analysis showed that mOS was different in R/MHNSCC patients with different apatinib lines and durations, Cox regression analysis did not show the same results.

Hypertension and hand-foot syndrome are common side effects of antiangiogenic drugs. In our study, except for one case of hospitalization in a patient with grade 3 oral ulcers, the severity of hypertension, hand-foot syndrome, fatigue, oral ulcers, and nausea and vomiting were all grade 1 or grade 2, with an incidence of 39.6%, 32.1%, 32.1%, 28.3% and 20.8% respectively. There were only 2 cases of grade 3/4 adverse reactions. Compared with previous clinical studies of apatinib for the treatment of recurrent or metastatic nasopharyngeal carcinoma, our study demonstrated a similar incidence of adverse reactions, but the incidence of proteinuria in our study was significantly lower. As the other three studies used a higher starting dose of apatinib (500 mg), it is possible that the development of proteinuria is closely related to drug dose^34–36^. According to the results of our study, compared with cetuximab and platinum combined with 5-FU chemotherapy, apatinib has a similar incidence of adverse reactions, but the incidence of grade 3 adverse reactions is significantly lower^4,37^. In addition, apatinib is associated with a significantly lower incidence of adverse reactions than afatinib, which is another second-line therapy. Nevertheless, the incidence of adverse reactions with apatinib is still higher compared to pembrolizumab^24,38^.

This study has some limitations. First, this study only included patients in Jilin, Heilongjiang, and Liaoning provinces of China, and was a retrospective study of a single ethnicity. Second, patients who had received at least first-line therapy were included. Therefore, patients often receive other treatments while receiving apatinib, including traditional Chinese medicine. These regular or irregular treatments have some impact on the PFS and OS of patients. Third, this study is a single-arm small sample study, and a large randomized controlled trial is needed to explore the efficacy and safety of apatinib.

## Conclusion

The result of our study shows that apatinib mesylate has good efficacy in inoperable R/M HNSCC as a second-line and above treatment. The most common adverse reactions occurred in patients were hypertension, hand-foot syndrome, fatigue, mouth ulcers, nausea and vomiting, which were tolerable and manageable.

## Data Availability

All data generated or analyzed during this study are included in this published article. The datasets used or analyzed during the current study are available from the corresponding author on reasonable request.
